# Aligning to the sample-specific reference sequence to optimize the accuracy of next-generation sequencing analysis for hepatitis B virus

**DOI:** 10.1007/s12072-015-9645-x

**Published:** 2015-07-25

**Authors:** Wen-Chun Liu, Chih-Peng Lin, Chun-Pei Cheng, Cheng-Hsun Ho, Kuo-Lun Lan, Ji-Hong Cheng, Chia-Jui Yen, Pin-Nan Cheng, I-Chin Wu, I-Chen Li, Bill Chia-Han Chang, Vincent S. Tseng, Yen-Cheng Chiu, Ting-Tsung Chang

**Affiliations:** Department of Internal Medicine, National Cheng Kung University Hospital, College of Medicine, National Cheng Kung University, 138 Sheng-Li Road, Tainan, 70403 Taiwan; Infectious Disease and Signaling Research Center, National Cheng Kung University, Tainan, Taiwan; Yourgene Bioscience, Taipei, Taiwan; Department of Computer Science and Information Engineering, National Cheng Kung University, Tainan, Taiwan

**Keywords:** Alignment stage, Coverage, Divergence, Single nucleotide variants

## Abstract

**Background:**

Hepatitis B virus (HBV) quasispecies are crucial in the pathogenesis of chronic liver disease. Next-generation sequencing (NGS) is powerful for identifying viral quasispecies. To improve mapping quality and single nucleotide variant (SNV) calling accuracy in the NGS analysis of HBV, we compared different mapping references, including the sample-specific reference sequence, same genotype sequences and different genotype sequences, according to the sample.

**Methods:**

Real Illumina HBV datasets from 86 patients, and simulated datasets from 158 HBV strains in the GenBank database, were used to assess mapping quality. SNV calling accuracy was evaluated using different mapping references to align Real Illumina datasets from a single HBV clone.

**Results:**

Using the sample-specific reference sequence as a mapping reference produced the largest number of mappable reads and coverages. With a different genotype mapping reference, the consensus sequence derived from the Real Illumina datasets of the single HBV clone showed 21 false SNV callings in polymerase and surface genes, the regions most divergent between the mapping reference and this HBV clone. A ~6 % coverage of most of these false SNVs was yielded even with a same genotype mapping reference, but none with the sample-specific reference sequence.

**Conclusions:**

Using sample-specific reference sequences as a mapping reference in NGS analysis optimized mapping quality and the SNV calling accuracy for HBV quasispecies.

**Electronic supplementary material:**

The online version of this article (doi:10.1007/s12072-015-9645-x) contains supplementary material, which is available to authorized users.

## Introduction

Next generation sequencing (NGS), also known as ultra-high throughput sequencing, is a powerful tool for discovering diseases with novel mutations and for detecting traces of pathogenic microorganisms [1, 2]. It has also been used for sequencing human and microbial genomes and for identifying species.

Hepatitis B virus (HBV) infection causes a multitude of clinical conditions ranging from acute hepatitis to cirrhosis and hepatocellular carcinoma [3–5]. HBV is classified into genotypes A–J with an inter-genotypic divergence of at least 8 % in the complete genome sequence [6]. HBV genotypes B and C are prevalent in Taiwan [7]. Many studies have suggested that HBV genotypes show not only geographical distribution and ethnic specificity but are also associated with disease progression and responses to interferon treatment [8, 9]. HBV highly replicates its genome and daily releases more than 10^11^ virions. Because HBV viral polymerase reverse transcriptase (RT) has no proofreading activity, HBV has higher mutation rates than other DNA viruses and complex quasispecies [10]. Viral quasispecies have been implicated in the development of drug resistance, the exacerbation of chronic hepatitis B (CHB), and the development of liver cancer [10].

NGS has been used to characterize single nucleotide variations (SNVs) and their dynamics in HBV polymerase RT genes in serum from patients who had undergone antiviral treatment [11–13]. HBV sequence-reads from NGS were aligned to one reference sequence from either a consensus genotype sequence in public viral databases [12, 13] or a major viral sequence identified using polymerase chain reaction (PCR)-director sequencing [11]. However, the mapping reference for optimizing the results in NGS analysis has never been identified.

In this study, we used various types of mapping references: genotype sequences identical to and different from the NGS sample, and a sample-specific reference sequence derived from its NGS dataset to investigate the mapping quality and the accuracy of the SNV callings for the full HBV genome. We also illustrated the effect of divergence between the mapping reference and NGS datasets on mapping quality and the accuracy of the SNV callings.

## Materials and methods

### Patients

Informed consent was obtained from each patient. Eighty-six patients between the ages of 34 to 75 were enrolled. All patients were treatment-naïve and had serum HBV DNA ≥200,000 IU/mL and detectable hepatitis B surface antigen for at least 6 months. Patients with hepatitis C or D infection, human immunodeficiency virus infection, or autoimmune hepatitis were excluded. Serum samples of all the patients were stored at −80 °C until used. Clinical characteristics of the 52 genotype-C patients and 34 genotype-B patients are shown in Table 1.

**Table 1:** Characteristics of patients with different HBV genotypes

Variables	Genotype B (*n* = 34)	Genotype C (*n* = 52)	*p* value
Gender (m:f)	27:7	40:12	1.00
Age (years)	51.4 ± 9.5	51.9 ± 8.8	0.80
Albumin (g/dL)	4.3 ± 0.4	4.2 ± 0.4	0.19
AST (IU/L)	116.5 ± 131.2	172.4 ± 174.4	0.11
ALT (IU/L)	177.8 ± 186.8	214.9 ± 207.5	0.39
Creatinine (mg/dL)	0.9 ± 0.2	0.9 ± 0.2	0.53
Total bilirubin (mg/dL)	1.6 ± 4.0	1.1 ± 0.8	0.39
HBeAg (+/−)	5/29	14/38	0.28
HBV DNA (log10 IU/mL)	6.5 ± 1.4	6.6 ± 1.5	0.76
Cirrhosis (+/−)	7/27	22/30	0.06

Data of continuous variables are mean ± SD. *p* values for continuous variables and nominal variables are from two-tailed independent *t* tests and *χ* ^2^ tests, respectively

### Extracting and amplifying full HBV genomic DNA

HBV DNA from 200 μL of serum was extracted using the Viogene Blood and Tissue Extraction Mini DNA Extractor kit (Viogene BioTek, New Taipei City, Taiwan). Fragments of the HBV DNA full genome were amplified using PCR with nine primer sets (Supplementary Table 1) and High Fidelity DNA polymerase (Thermo Fisher Scientific, Pittsburgh, PA, USA). The PCR condition was 5 min at 94 °C followed by 40 cycles of 1 min at 94 °C, 1 min at 50 °C, and 1.5 min at 72 °C. All amplified PCR products were run in a 1 % agarose gel electrophoresis and were purified using the GEL/PCR Purification Mini Kit (Favorgen Biotech, Ping-Tung, Taiwan). Nine PCR fragments were mixed and were subjected to NGS.

### Viral genome sequencing using massively parallel NGS

Massively-parallel NGS with multiplexed tags was carried out using a genome analyzer, as previously described [11]. In brief, HBV DNA was fragmented using sonication and was cut into suitable sizes. These fragments were purified and were then end-repaired and A-tailed using DNA Polymerase I Klenow Fragment (3′ → 5′ exo-) (New England Biolabs, Ipswich, MA, USA). DNA fragments ligated with indexed adapters were amplified using 10–18 cycles of PCR reaction. The DNA library was quantified by Qubit fluorometer (Qubit dsDNA HS assay, Thermo Fisher Scientific, Life Technology) and real time PCR (KAPA Library Quantification Kit Illumina® platforms, KAPA biosystems). Experion Automated Electrophoresis System (Bio-Rad Laboratories, Hercules, CA, USA) was used to validate the size of the library. After it had been validated, the library was sequenced (HiSeq™ 2500; Illumina, San Diego, CA, USA).

### Genomic analysis of NGS data

For NGS high-throughput data, low-quality bases of raw reads were first trimmed using Seqtk (https://github.com/lh3/seqtk), which uses a modified Mott trimming algorithm. All parameters were default settings except maximally trimming down to 1 bp to remove as many low-quality bases as possible but still keeping paired-end information. At the alignment stage, the trimmed reads were then mapped to the mapping reference genome using BWA (BWA-MEM) [14] with a −M parameter setting and 16 threads to get correct and consistent mapping statistics. Because almost all genome mappers, like BWA, were designed for linear genomes, they are not well suited for circular genomes like HBV genomes, especially when reads spanning the end of the genome have worse mapping performance. Therefore, we manually concatenated the end parts, 600 bases, ranging from the beginning of the 5′ end to the 3′ end to avoid overhanging reads. The mapping results were then processed using SAMTools [15] to remove reads that mapped to multiple positions and reads that had poor mapping quality scores.

### Direct Sanger sequencing and NGS of HBV clones

Serum HBV DNA of two patients with CHB were extracted and amplified using PCR. The designed primers were modified [7] to amplify the full-length HBV genome and then were cloned into yT&A plasmid (Yeastern Biotech, Taipei, Taiwan). HBV full genomes of Clone_N6 (KJ790199) and Clone_H44 (KJ790200) were sequenced using a direct Sanger sequencer (Applied Biosystems, Life Technologies, Taipei, Taiwan). For NGS of the two HBV clones, the HBV full genomes in plasmids were amplified using primers with restriction site sapI [16], then self-ligated using T4 ligase to produce a circular form HBV genome, and amplified into nine fragments that were subjected to NGS as described in “Viral genome sequencing using massively parallel NGS”.

### Mapping references chosen at the alignment stage during NGS data analysis

To optimize the NGS data analysis, five different mapping references were used at the alignment stage to compare the mapping results. Four full-length HBV genome sequences, FJ787477 (genotype B, Asia), JN315779 (genotype C, Asia), KJ790200 (Clone_H44; genotype B, Taiwan), and KJ790199 (Clone_N6; genotype C, Taiwan) in the GenBank database were used. Furthermore, a sample-specific reference sequence, the consensus sequence obtained from the NGS reads of each sample through alignment with its same genotype mapping reference (FJ787477 or JN315779), was also used as a mapping reference sequence for the NGS datasets.

### HBV genotyping

The HBV genotype was determined using a melting curve analysis with LightCycler hybridization probes as previously described [17]. The derived consensus sequence of NGS reads were aligned to the standard full-length HBV genomes (genotype A–H) from the GenBank database to confirm the genotyping results. HBV genotype was identified using phylogenetic analysis software (Mega 6.0) [18].

### Evaluating the mapping quality of simulated datasets from 158 HBV strains in the GenBank database with the alignment to different genotype sequences

To evaluate the quality of mapping results with the alignment to different genotype references, 158 HBV complete genomes (34 genotype A, 33 genotype B, 39 genotype C, and 52 genotype D strains) with 4 common genotypes from the GenBank database were collected. The simulated NGS reads were produced from each strain using sequence alignment/mapping software (SAMtools wgsim) [15]. To analyze these 158 simulated NGS datasets, the full genome of each HBV strain was also used as a mapping reference for alignment, respectively. The mapping results—mappable reads, properly paired reads, broken paired reads, and singleton reads—were evaluated.

### Calculating nucleotide divergence

Nucleotide divergences were calculated using DNA sequence polymorphism software (DNasp 5.10.1) [19]. One hundred sites for window length were set when we calculated the divergences.

### Statistical analysis

Continuous variables were compared using Student’s *t* test for two independent groups. The changes in mapping quality between different mapping references were compared using paired *t* tests. The frequencies and distributions of categorical variables were compared using the Chi square tests or Fisher’s exact tests.

## Results

### The mapping quality of the NGS dataset was optimized when using a sample-specific reference sequence as the mapping reference

We compared the mapping quality of real Illumina datasets of viral genomes from 52 patients with genotype C HBV and 34 patients with genotype B HBV using different mapping references. The sample-specific reference sequence had the best quality, followed by the Taiwanese strain with the same genotype, the Asian strain with the same genotype, the Taiwanese strain with a different genotype, and the Asian strain with a different genotype (Table 2; Supplementary Table 2). In patients with genotype C HBV, the sample-specific reference sequence had the best mapping quality: the largest number of mappable reads, properly paired reads, and broken paired reads, and the lowest number of singletons. In addition, using a sample-specific sequence as a reference yielded the largest average coverages (38,362 ± 75,502), minimum coverages, and maximum coverages per nucleotide, and the smallest number of nucleotides with fewer than 30 coverages. Moreover, when using a sample-specific sequence, 97 % of the nucleotides in the full genome had more than 1000 coverages.

**Table 2:** Mapping NGS datasets of genotype C patients (*n* = 52) to different references of HBV full genome

Variables	FJ787477 (Geno. B, Asia)	KJ790200 (Geno. B, Taiwan)	JN315779 (Geno. C, Asia)	KJ790199 (Geno. C, Taiwan)	Sample specific reference
Mappable reads (%)	86.07 ± 9.13***	86.96 ± 8.94***	89.08 ± 9.06***	89.30 ± 8.95***^a^	89.41 ± 8.97
Properly paired reads (%)	83.28 ± 9.07***	84.41 ± 8.89***	86.65 ± 9.26	86.81 ± 9.13	86.89 ± 9.16
Broken paired reads (%)	0.99 ± 0.91**	0.99 ± 0.89**	1.50 ± 1.27	1.51 ± 1.28	1.75 ± 1.88
Singleton (%)	1.80 ± 0.76***	1.56 ± 0.67***	0.93 ± 0.59***	0.98 ± 0.58***	0.77 ± 0.56
Minimum coverage per nucleotide	93 ± 523***	277 ± 782***	1439 ± 2693	1471 ± 2698	1521 ± 2965
Maximum coverage per nucleotide	190,649 ± 454,110	192,928 ± 461,547	195,836 ± 475,955	197,166 ± 476,190	197,173 ± 475,579
Average coverage per nucleotide	33,833 ± 64,303**	34,214.4 ± 65,765**	38,287 ± 75,400	38,335 ± 75,419^a^	38 362.4 ± 75,502
Nucleotides covered <30 (%)	0.85 ± 1.16***	0.35 ± 1.10**	0.23 ± 1.12	0.24 ± 1.15	0.22 ± 1.07
Nucleotides covered >1000 (%)	93.69 ± 5.23***	94.85 ± 4.99***	97.15 ± 4.40***	97.17 ± 4.40	97.19 ± 4.40

Total reads after quality trimming = 1,504,374 ± 2,780,326; data are mean ± SD; FJ787477, KJ790200, JN315779, and KJ790199 were from GenBank database; *Geno.* genotype; sample-specific reference was from the NGS reads aligned to JN315779; *p* values for differences between samples-specific reference and each reference from the GenBank database (*p < 0.05; **p < 0.01; ***p < 0.001) and for differences between JN315779 and KJ790199 (^a^ *p* < 0.01) are from two-tailed independent *t* tests

Using a Taiwanese genotype C mapping reference yielded larger mappable reads (*p* < 0.0001) and average coverage per nucleotide (*p* < 0.0001) than did using the Asian genotype C mapping reference. A similar pattern of mapping quality was detected in NGS datasets from 34 genotype B patients aligned to different HBV mapping references (Supplementary Table 2).

### Simulated NGS datasets for which a sample-specific sequence was used as a reference yielded improved mapping quality

Simulated NGS reads were produced from 158 HBV full genomes (genotypes A–D HBV) obtained from the GenBank database using the Wgsim read simulator [15]. To analyze these simulated NGS datasets, each full genome was also used as mapping reference for alignment. The mapping qualities categorized by subgenotypes were shown in Supplementary Table 3. The improved mapping qualities of simulated NGS datasets aligned to the same genotype sequence, especially the same subgenotype sequence in genotype B, showed a higher percentage of mappable reads and properly paired reads and a lower percentage of singletons than did datasets aligned to different genotypes.

### Datasets aligned to different genotypes yielded false SNVs in the consensus sequence derived from NGS reads of a single HBV clone

**Fig. 1:**
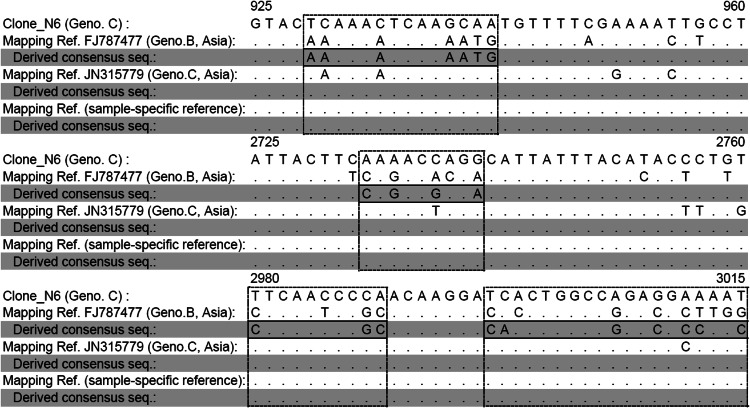
A comparison of different mapping reference sequences and their derived consensus sequences of NGS reads from Clone_N6 (Genotype C) with the direct sequence of Clone_N6. Asian genotypes B (GenBank accession number FJ787477) and C (GenBank accession number JN315779) from the NCBI GenBank database. The sample-specific reference was a consensus sequence obtained from Clone_N6 NGS reads aligned to JN315779. The derived consensus sequences were obtained from Clone_N6 NGS reads with alignment against their preceding mapping references, respectively. The *thick lines* indicate false SNVs in the derived consensus sequence

The mapping results of NGS reads from a Taiwanese genotype C HBV Clone_N6 using different mapping references: sample-specific reference sequence JN315779 (Genotype C, Asia) and reference sequence FJ787477 (Genotype B, Asia) were assessed. When compared with the sequence of Clone_N6 derived from direct sequencing, the consensus sequence contained 21 false SNVs when using a different genotype mapping reference (Fig. 1). Consensus sequences derived from the same dataset were identical to the sequence of Clone_N6 using either the same genotype reference or a sample-specific reference sequence as mapping references.

### Regions of high divergence between single HBV clone and mapping references involved the false SNVs of NGS analysis

**Fig. 2:**
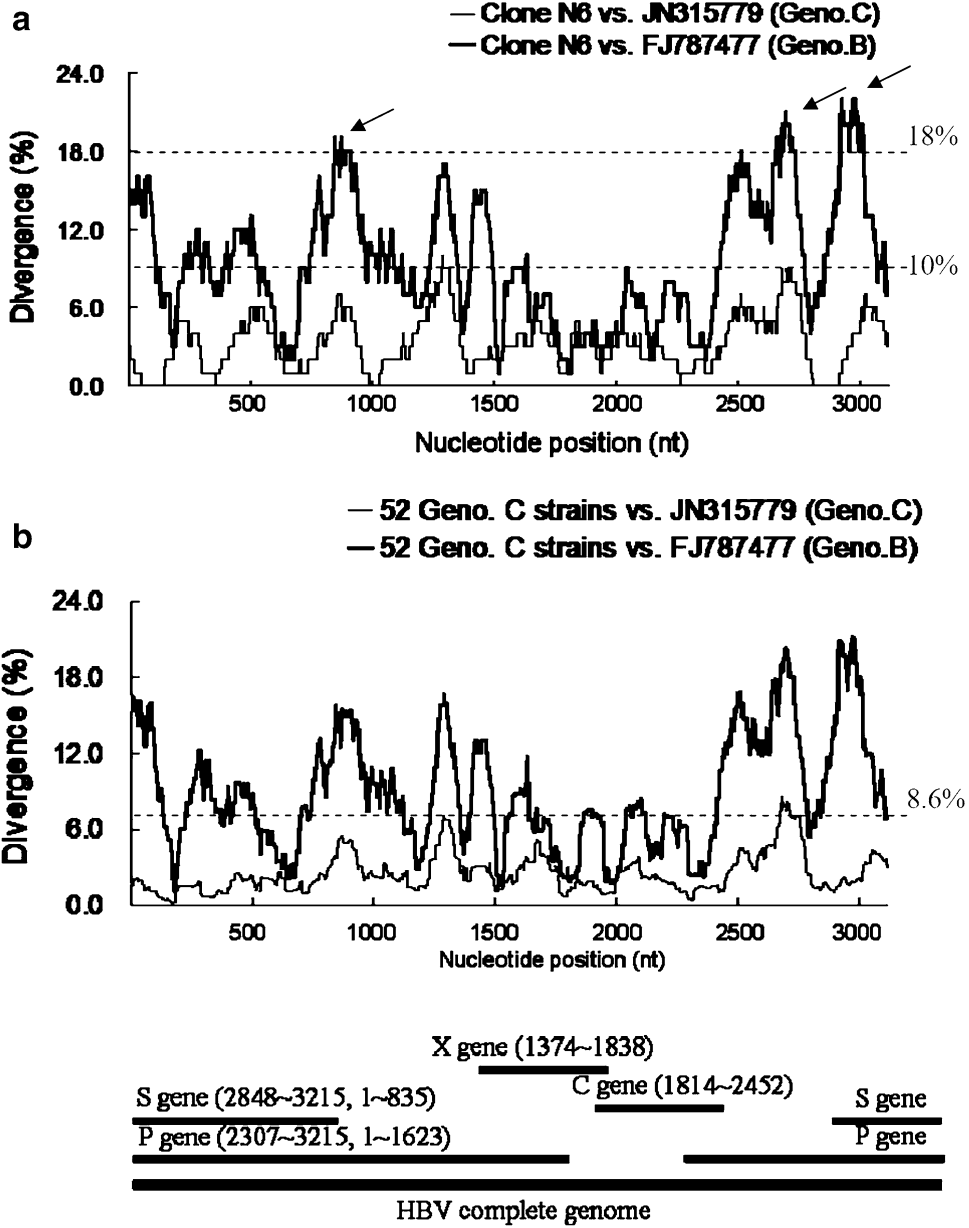
**a** Sequence divergence between Clone_N6 (genotype C) and mapping references, FJ787477 (genotype B, Asia) and JN315779 (genotype C, Asia), respectively. *Arrows* indicate three regions with the highest divergence over 18 % at nt929–942, nt2733–2741, and nt2980–3015. **b** Mean sequence divergence between derived consensus sequences from NGS reads of 52 patients with genotype C chronic hepatitis B and mapping references, FJ787477 (genotype B, Asia) and JN315779 (genotype C, Asia). A schematic diagram of the hepatitis B virus complete genome and four genes are shown in the bottom panel and the positions correspond to the *x*-axis of (**a**, **b**)

The percentage of mappable reads from the Clone_N6 NGS dataset was 98.7 using a sample-specific reference sequence, 97.0 % using JN315779, and 96.8 % using FJ787477. The coverage and percentage of false SNVs in the consensus sequences derived from Clone_N6 (genotype C) NGS reads when aligned to different HBV mapping references are shown in Table 3. When the NGS reads were aligned to a different genotype strain (FJ787477), the derived consensus sequence showed 21 false SNVs with 2–3 log reductions of coverage and an enormous change in nucleotide percentages. Interestingly, these false SNVs were located exactly at the three highest divergence regions, viz., nt929–942 (P gene/RT domain), nt2733–2741 (P gene/terminal protein domain), and nt2980–3015 (P gene/spacer domain; pre-S1 region), with a divergence of >18 % between Clone_N6 (genotype C) and the mapping reference FJ787477 (Genotype B, Asia) (Fig. 2a).

**Table 3:** Coverage and percentage of false SNVs of NGS reads (Clone_N6, genotype C) with alignment to different references of HBV full genome

NT	N6 sequence/false SNVs	Sample-specific reference	JN315779 (Geno. C, Asia)^a^	FJ787477 (Geno. B, Asia)^a^
929	T/A	586,307/229 (99.8/0.1)	17,271/456 (97.3/2.6)	116/449 (20.5/79.5)
930	C/A	578,003/378 (99.8/0.1)	17,200/425 (97.4/2.4)	62/402 (13.2/85.4)
934	C/A	542,825/281 (99.9/0.1)	17,234/395 (97.7/2.2)	75/367 (17.0/83.0)
939	G/A	505,379/307 (99.9/0.1)	17,548/59 (99.7/0.0)	10/407 (2.4/97.6)
940	C/A	498,076/149 (99.9/0.0)	17,530/76 (99.6/0.0)	12/402 (2.9/97.1)
941	A/T	493,990/49 (99.9/0.0)	11,199/325 (97.2/0.1)	12/300 (3.8/95.9)
942	A/G	489,435/248 (99.9/0.1)	15,823/424 (97.4/0.1)	23/304 (7.0/93.0)
2733	A/C	729,923/160 (99.9/0.0)	61,679/193 (99.7/0.3)	41/115 (26.3/73.7)
2735	A/G	720,464/432 (99.9/0.1)	49,152/393 (99.2/0.8)	25/121 (17.1/82.9)
2738	C/G	700,914/218 (99.9/0.0)	49,206/200 (99.5/0.4)	25/103 (18.7/76.9)
2741	G/A	683,135/889 (99.9/0.1)	48,724/789 (98.4/1.6)	29/124 (19.0/81.1)
2980	T/C	739,842/2381 (99.7/0.3)	421,040/2437 (99.4/0.6)	1308/2207 (37.2/62.8)
2988	C/G	756,854/793 (99.8/0.1)	33,655/1712 (95.1/4.8)	884/1670 (34.5/65.3)
2989	A/C	757,643/834 (99.8/0.1)	33,597/1762 (95.0/5.0)	844/1718 (32.9/67.0)
2997	T/C	752,699/1392 (99.8/0.2)	32,840/2108 (94.0/6.0)	415/1810 (18.7/81.4)
2998	C/A	754,680/846 (99.8/0.1)	33,348/1753 (95.0/5.0)	758/1357 (35.8/64.1)
3006	A/G	730,064/1055 (99.8/0.1)	32,595/1863 (94.6/5.4)	25/1416 (1.7/98.1)
3009	G/C	718,234/667 (99.9/0.1)	32,503/1615 (95.1/4.7)	1/1296 (0.1/99.8)
3011	A/C	708,480/967 (99.8/0.1)	32,393/1688 (95.0/4.9)	0/1266 (0.0/100.0)
3012	A/C	705,693/646 (99.9/0.1)	32,481/1596 (95.3/4.7)	0/941 (0.0/99.8)
3015	T/C	691,698/806 (99.9/0.1)	32,366/1695 (95.0/5.0)	1/946 (0.1/96.4)

Data are coverage (%); sample-specific reference was from the NGS reads with alignment to JN315779

*NT* nucleotide, *SNV* single nucleotide variation, *Geno.* genotype

^a^FJ787477 and JN315779 were from the GenBank database

Using a mapping reference with the same genotype (JN315779) for Clone_N6 NGS reads, even the derived consensus sequence was identical to the Clone_N6 sequence, and decades of SNVs were still detected with the coverage percentage up to 6 % (Table 3). The sequence divergences of each sliding window between Clone_N6 and mapping references with JN315779 were all below 10 % in the HBV full genome (Fig. 2a). Using a mapping reference with the sample-specific reference sequence showed the best results of NGS analysis, with a percentage of false SNVs <0.3. This false SNV rate was considered mismatch error values and could be ignored in NGS analysis [11]. Furthermore, we found 14 false SNVs in the consensus sequence derived from Clone_H44 (genotype B) NGS reads when mapping to reference JN315779 (Genotype C, Asia). All the false SNVs located in nt940–942 (P gene/RT domain), nt2759–2775 (P gene/terminal protein domain), and nt2783–2790 (P gene/terminal protein domain), showed high divergence (>15 %) between Clone_H44 and JN315779 (Supplementary Fig. 1A).

### High prevalence of SNVs in 52 patients associated with a high divergence between their derived HBV consensus sequences and mapping references

We obtained all the sequence divergences of each sliding window by comparing each HBV-derived consensus sequence from 52 patients infected with genotype C HBV with mapping references JN315779 (Genotype C, Asia) and FJ787477 (Genotype B, Asia). Mean sequence divergences of genotype C consensus strains relative to two different genotype mapping references were shown as two demarcating curves (Fig. 2b). The profiles were similar to those in Fig. 2a. When using FJ787477 as a mapping reference, all the mean sequence divergences of each window except those within nt2137–2250 of the core gene were significantly higher than those referenced to JN315779 (*p* < 0.0001).

Interestingly, the derived consensus sequence at 94 nucleotide positions of the HBV full genome in at least one of 52 patients with genotype C HBV contained inconsistent variants when using different genotype mapping references (Supplementary Table 4). Seventeen inconsistent variants occurred in more than 20 % and ten inconsistent variants in more than 40 % of these patients (Table 4). These inconsistent SNVs were located at high divergence regions (over 13 %): nt939–942, nt1353–1362, and nt2980–3015, and they were probably false SNVs (Fig. 2b). In addition, mean sequence divergences between 34 genotype B-derived consensus sequences from CHB patients and each of two different genotype mapping references are shown as two demarcating curves in Supplementary Fig. 2A, in which the profiles are similar to those in Fig. 2b. When comparing different genotype mapping references, 111 nucleotides in the HBV full genome of derived consensus sequences expressed inconsistent variants in at least one patient (Supplementary Table 5). When using JN315779 as a mapping reference, all the mean sequence divergences except those within nt368–481, nt685–790, nt1207–1313, nt1802–2000, and nt2133–2584 were significantly higher than those referenced to FJ787477 (*p* < 0.0001) (Supplementary Fig. 1B).

**Table 4:** Prevalence of probable false SNVs of genotype C patients (*n* = 52) to different references of HBV full genome

NT	Mapping reference (Geno. C/B)	Inconsistent SNVs comprising derived consensus sequences	Number of patients (%)
		JN315779 → FJ787477 (Geno. C, Asia) (Geno. B, Asia)	
939	G/A	G → A	38 (73.1)
940	C/A	C → A	38 (73.1)
941	A/T	A → T	27 (51.9)
942	A/G	A → G	23 (44.2)
1353	T/C	T → C	15 (28.8)
1356	G/C	G → C	15 (28.8)
1359	A/G	A → G	13 (25.0)
1362	C/T	C → T	12 (23.1)
2980	T/C	T → C	17 (32.7)
2988	C/G	C → G	18 (34.6)
2989	A/C	A → C	20 (38.5)
2997	T/C	T → C	25 (48.1)
2998	C/C	C → A	22 (42.3)
3006	A/G	A → G	26 (50.0)
3009	G/C	G(29)/A(2) → C	31 (59.6)
3012	A/T	A → C	31 (59.6)
3015	T/G	T → C(23)/G(17)	40 (76.9)

Inconsistent SNVs comprising derived consensus sequences from at least 20 % of patients are shown

*SNV* single nucleotide variation, *NT* nucleotide, *Geno.* genotype

### The mean divergence of any consecutive 100-nucleotide segments in genotypes A–D

**Fig. 3:**
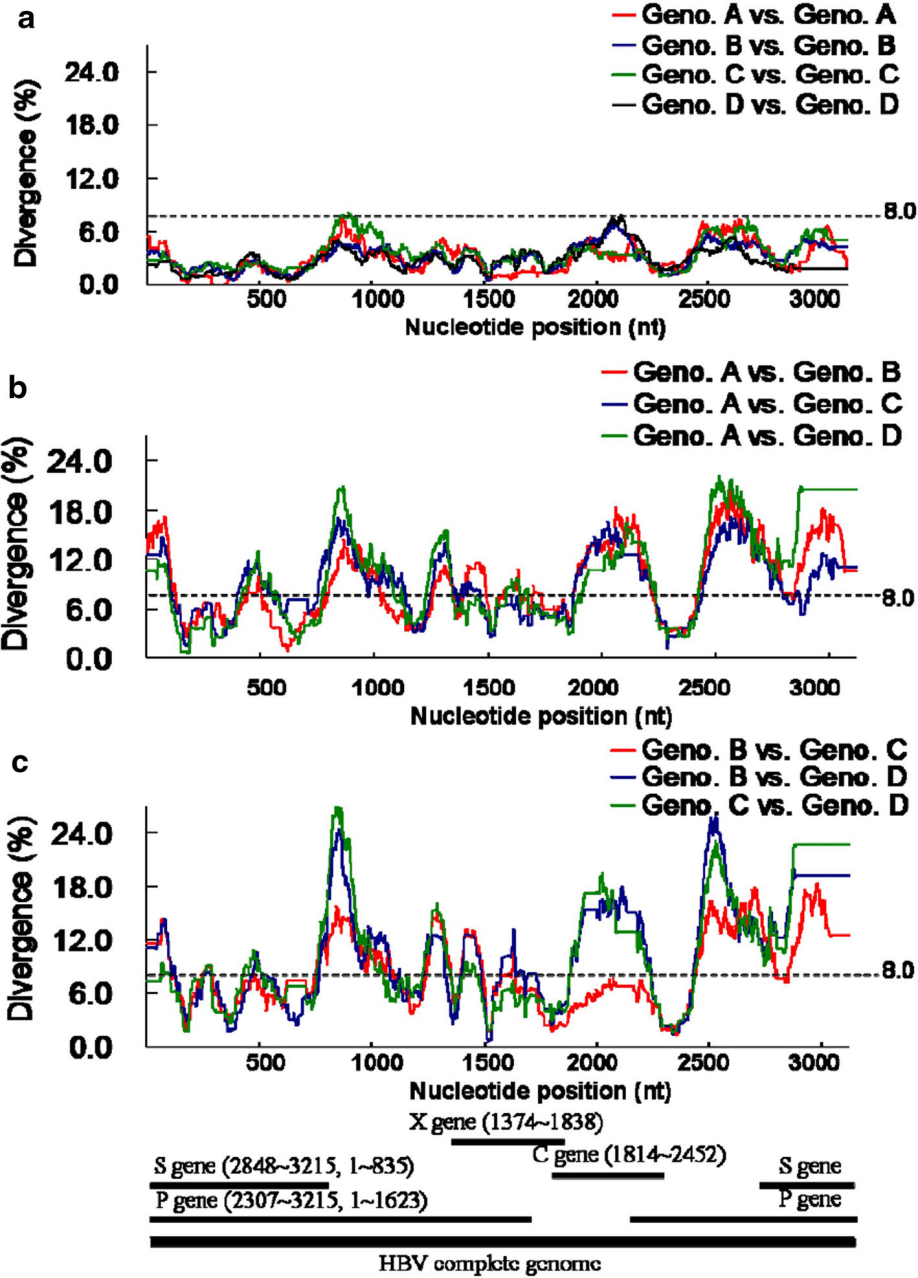
Comparison of mean sequence divergences between different hepatitis B virus (HBV) genotype populations. A total of 158 HBV strains were collected from the GenBank database (34 genotype A, 33 genotype B, 39 genotype C, and 52 genotype D) and analyzed. **a** Mean sequence divergences within the same genotype were expressed. **b**, **c** Mean sequence divergences between different genotypes were expressed. A schematic diagram of the hepatitis B virus complete genome and four genes are shown in the bottom panel and the positions correspond to the *x*-axis of (**a**–**c**). *Geno. A* genotype A, *Geno. B* genotype B, *Geno. C* genotype C, *Geno. D* genotype D

We collected 158 HBV full genomes (genotypes A–D) from the GenBank database. Within the same genotype, the mean divergence of any consecutive 100-nucleotide segment was ≤8.0 % (Fig. 3). Between any two different genotypes, there were high divergences in most regions of the HBV full genome, except the core gene between genotypes B and C.

## Discussion

Various NGS platforms, including the Illumina HiSeq, Illumina Genome Analyzer, Illumina Miseq, Roche 454, and SOLiD4, differ in engineering configurations and sequencing chemistry. In the present study, we used the Illumina Hiseq 2500 system to analyze HBV full genome in viral quasispecies; it has the advantages of short run times, long read lengths, and high data quality. With an average read length of 110 bp and an average coverage ≥25,000 after quality trimming, the depth and quality of the sequencing results are admissible for analyzing viral quasispecies.

At the alignment stage, mapping short reads against a reference genome is typically the first step in analyzing such NGS data, and it should be as accurate as possible. The mapping reference is crucial for mapping quality and for the quality of the SNV calls. Previous HBV-related NGS analyses used the consensus genotype sequence [12, 13] or a sequence from direct sequencing of PCR products [11] as mapping references. This is the first study to compare the mapping quality and the accuracy of the SNV calls for NGS analysis of the HBV full genome using different mapping references. When a sample-specific reference sequence was used, the mappable reads were 89.4 % of total reads with highest properly paired reads and broken paired reads and lowest singleton reads, which indicated that mapping quality was substantially improved. Coverage is important for the quality of SNV calls. High coverage regions or bases tend to have a higher call quality. Using a sample-specific reference sequence as mapping reference increased the average coverage for each nucleotide. When simulated NGS datasets were aligned to the same genotype sequence, especially the same subgenotype sequence in genotype B or from the same country, mapping qualities were also improved.

HBV quasispecies are importantly implicated in the pathogenesis of chronic liver diseases. For example, several mutations of the HBV genome are crucial for developing HCC and cirrhosis [20–23]. Therefore, accurate SNV calls are important for analyzing the HBV full genome in NGS. Along with the verification using cloned sequences, we identified numerous false SNVs in a major strain when mapping with different viral genotypes to the preceding sample (Table 3). These false SNV calls were contributed by fewer mappable reads, a profound reduction in coverages, and the change of nucleotide composition at one site. In addition, we also confirmed that there were probably many false SNVs in derived consensus sequences from patients when using a different viral genotype as a mapping reference (Table 4). More than 20 % of the patients had numerous false SNVs, particularly in high-divergence regions (Table 4; Supplementary Tables 4, 5), which suggested that a different genotype sequence was an inappropriate mapping reference for HBV NGS analysis.

HBV mutants have been demonstrated in patients with acute fulminant or chronic infection [10]. Mutations in the RT region of viral P gene have been associated with the resistance to antiviral drugs [24, 25]. Mutations on the PreS1 or PreS2 promoter were correlated with the occurrence of HCC [26, 27]. While using different genotype mapping references, we found that false SNV calls of major strains were located in high-divergence regions, such as the P-gene/RT domain, P-gene/terminal protein domain, and preS1 region in the HBV genome for genotypes A–D (Fig. 2). We suggest using a sample-specific reference sequence as a mapping reference for NGS analysis in studies of P-gene variants for drug-resistance [24, 25] and S-gene variants for the pathogenesis of HCC [26, 27]. Emerging evidence supports the notion that certain drug-resistant HBV minor strains are crucial for the progression of liver diseases and are predictors of subsequent treatment failure [23, 28]. They may accumulate and eventually dominate under a long-term selection effect during antiviral treatment [23].

By taking advantage of an abundance of genetic information when using NGS, more accurate and detailed SNVs of HBV quasispecies can be obtained than with previous methods, such as INNO-LiPA and clonal HBV sequencing. However, even when we used the same genotype sequence as a mapping reference, decades of false SNVs still existed with a coverage up to 6 % (Table 3). Using a mapping reference with a sample-specific reference sequence yielded the best NGS analysis results with a coverage percentage of false SNVs <0.3 %, which was considered a mismatch error value and could be ignored for NGS analysis in the Illumina platform [11]. Therefore, using a sample-specific reference sequence to align the NGS dataset is crucial for accurate SNV calls of viral quasispecies.

In conclusion, we conclude that a sample-specific reference sequence, which provides the highest mapping quality and SNV call accuracy, should be used as the mapping reference in the NGS analysis of emerging HBV variants, especially for the studies of the P gene and S gene.

## Electronic supplementary material

Supplementary material 1 (PDF 214 kb)

## Abbreviations

CHB: Chronic hepatitis B
DNasp: DNA sequence polymorphism
Geno.: Genotype
HBV: Hepatitis B virus
NGS: Next generation sequencing
NT: Nucleotide
PCR: Polymerase chain reaction
SNV: Single nucleotide variant

## References

[1] Ley TJ, Mardis ER, Ding L, Fulton B, McLellan MD, Chen K (2008). DNA sequencing of a cytogenetically normal acute myeloid leukaemia genome. Nature.

[2] Isakov O, Modai S, Shomron N (2011). Pathogen detection using short-RNA deep sequencing subtraction and assembly. Bioinformatics.

[3] Lok AS, McMahon BJ (2007). Chronic hepatitis B. Hepatology.

[4] EASL clinical practice guidelines (2012). Management of chronic hepatitis B virus infection. J Hepatol.

[5] Liaw YF, Chu CM (2009). Hepatitis B virus infection. Lancet.

[6] Liu CJ, Kao JH (2013). Global perspective on the natural history of chronic hepatitis B: role of hepatitis B virus genotypes A–J. Semin Liver Dis.

[7] Liu WC, Phiet PH, Chiang TY, Sun KT, Hung KH, Young KC (2007). Five subgenotypes of hepatitis B virus genotype B with distinct geographic and virological characteristics. Virus Res.

[8] Kidd-Ljunggren K, Miyakawa Y, Kidd AH (2002). Genetic variability in hepatitis B viruses. J Gen Virol.

[9] Nie JJ, Sun KX, Li J, Wang J, Jin H, Wang L (2012). A type-specific nested PCR assay established and applied for investigation of HBV genotype and subgenotype in Chinese patients with chronic HBV infection. Virol J.

[10] Blum HE (1993). Hepatitis B virus: significance of naturally occurring mutants. Intervirology.

[11] Nishijima N, Marusawa H, Ueda Y, Takahashi K, Nasu A, Osaki Y (2012). Dynamics of hepatitis B virus quasispecies in association with nucleos(t)ide analogue treatment determined by ultra-deep sequencing. PloS ONE.

[12] Margeridon-Thermet S, Shulman NS, Ahmed A, Shahriar R, Liu T, Wang C (2009). Ultra-deep pyrosequencing of hepatitis B virus quasispecies from nucleoside and nucleotide reverse-transcriptase inhibitor (NRTI)-treated patients and NRTI-naive patients. J Infect Dis.

[13] Solmone M, Vincenti D, Prosperi MC, Bruselles A, Ippolito G, Capobianchi MR (2009). Use of massively parallel ultradeep pyrosequencing to characterize the genetic diversity of hepatitis B virus in drug-resistant and drug-naive patients and to detect minor variants in reverse transcriptase and hepatitis B S antigen. J Virol.

[14] Li H, Durbin R (2009). Fast and accurate short read alignment with Burrows–Wheeler transform. Bioinformatics.

[15] Li H, Handsaker B, Wysoker A, Fennell T, Ruan J, Homer N (2009). The sequence alignment/map format and SAMtools. Bioinformatics.

[16] Gunther S, Li BC, Miska S, Kruger DH, Meisel H, Will H (1995). A novel method for efficient amplification of whole hepatitis B virus genomes permits rapid functional analysis and reveals deletion mutants in immunosuppressed patients. J Virol.

[17] Liu WC, Mizokami M, Buti M, Lindh M, Young KC, Sun KT (2006). Simultaneous quantification and genotyping of hepatitis B virus for genotypes A–G by real-time PCR and two-step melting curve analysis. J Clin Microbiol.

[18] Tamura K, Stecher G, Peterson D, Filipski A, Kumar S (2013). MEGA6: molecular evolutionary genetics analysis version 6.0. Mol Biol Evol.

[19] Librado P, Rozas J (2009). DnaSP v5: a software for comprehensive analysis of DNA polymorphism data. Bioinformatics.

[20] Chen YM, Wu SH, Qiu CN, Yu DJ, Wang XJ (2013). Hepatitis B virus subgenotype C2- and B2-associated mutation patterns may be responsible for liver cirrhosis and hepatocellular carcinoma, respectively. Braz J Med Biol Res.

[21] Lin CL, Kao JH (2008). Hepatitis B viral factors and clinical outcomes of chronic hepatitis B. J Biomed Sci.

[22] Park YM, Jang JW, Yoo SH, Kim SH, Oh IM, Park SJ (2014). Combinations of eight key mutations in the X/preC region and genomic activity of hepatitis B virus are associated with hepatocellular carcinoma. J Viral Hepat.

[23] Singla B, Chakraborti A, Sharma BK, Kapil S, Chawla YK, Arora SK (2013). Hepatitis B virus reverse transcriptase mutations in treatment Naive chronic hepatitis B patients. J Med Virol.

[24] Song ZL, Cui YJ, Zheng WP, Teng DH, Zheng H (2012). Diagnostic and therapeutic progress of multi-drug resistance with anti-HBV nucleos(t)ide analogues. World J Gastroenterol.

[25] Yim HJ, Hwang SG (2013). Options for the management of antiviral resistance during hepatitis B therapy: reflections on battles over a decade. Clin Mol Hepatol.

[26] Li GJ, Harrison TJ, Yang JY, Chen QY, Wang XY, Fang ZL (2013). Combined core promoter mutations and pre-S deletion of HBV may not increase the risk of HCC: a geographical epidemiological study in Guangxi, China. Liver Int.

[27] Qu L, Kuai X, Liu T, Chen T, Ni Z, Shen X (2013). Pre-S deletion and complex mutations of hepatitis B virus related to young age hepatocellular carcinoma in Qidong, China. PloS ONE.

[28] Bhattacharya D, Lewis MJ, Lassmann B, Phan T, Knecht G, Bickel M (2013). Combination of allele-specific detection techniques to quantify minority resistance variants in hepatitis B infection: a novel approach. J Virol Methods.

