# Supplementary material for: Aligning to the sample-specific reference sequence to optimize the accuracy of next-generation sequencing analysis for hepatitis B virus
Source: Hepatol Int. 2015 Jul 25;10(1):147–57. doi: 10.1007/s12072-015-9645-x (PMC4722079; doi:10.1007/s12072-015-9645-x)
Supplement: Supplementary file 1 — Supplementary material 1 (PDF 214 kb) [file 12072_2015_9645_MOESM1_ESM.pdf]

Supplementary Material

Figure Legends

Figure S1. (A) Sequence divergence between Clone_H44 (genotype B) and mapping references, FJ787477 (genotype B, Asia) and JN315779 (genotype C, Asia), respectively. (B) Mean sequence divergence between derived consensus sequences from NGS reads of 34 patients with genotype B chronic hepatitis B and mapping references, FJ787477 (genotype B, Asia) and JN315779 (genotype C, Asia), respectively. A schematic diagram of hepatitis B virus complete genome and four genes are shown and the position correspond to the *x*-axis of Figure A and B.

Figure S1

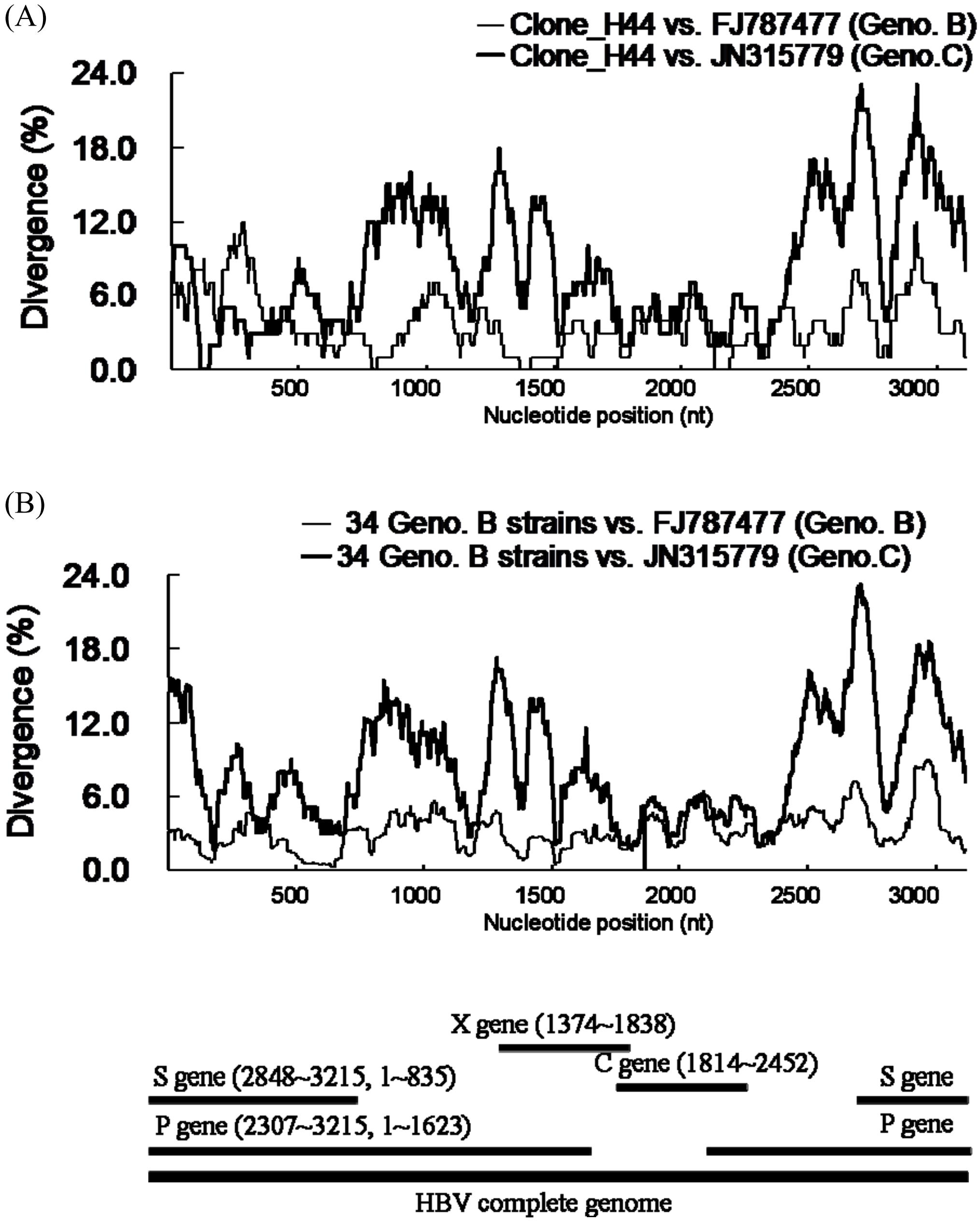
(A)
Clone_H44 vs. FJ787477 (Geno. B)
Clone_H44 vs. JN315779 (Geno.C)
Divergence (%)
24.0
18.0
12.0
6.0
0.0
500
1000
1500
2000
2500
3000
Nucleotide position (nt)
(B)
34 Geno. B strains vs. FJ787477 (Geno. B)
34 Geno. B strains vs. JN315779 (Geno.C)
Divergence (%)
24.0
18.0
12.0
6.0
0.0
500
1000
1500
2000
2500
3000
Nucleotide position (nt)
X gene (1374~1838)
C gene (1814~2452)
S gene (2848~3215, 1~835)
S gene
P gene (2307~3215, 1~1623)
P gene
HBV complete genome

Table S1. Primer sets for amplifying full HBV genome in NGS analysis

	Primers	Positions	Sequences	Product sizes
a.	Forward:	11-31	5'-TTCCACCAAGCTCTGCWAGAT-3'	547 bp
	Reverse:	536-557	5'-GAAACATAGAGGTTCCTTGAGC-3'	
b.	Forward:	374-397	5'-TGGATGTGTCTGCGGCGTTTTATC-3'	466 bp
	Reverse:	816-839	5'-GGGTTTAAATGTATACCCAAAGAC-3'	
c.	Forward:	710-728	5'-CTTTCCCCCACTGTYTGGC-3'	487 bp
	Reverse:	1176-1196	5'-GTTGCGTCAGCAAACACTTGG-3'	
d.	Forward:	1099-1119	5'-TCGCCAACTTACAAGGCCTTT-3'	508 bp
	Reverse:	1587-1606	5'-CGTGCAGAGGTGAAGCGAAG-3'	
e.	Forward:	1264-1283	5'-GATCCATACTGCGGAACTCC-3'	489 bp
	Reverse:	1735-1752	5'-CTCCTCCCCCAACTCCTC-3'	
f.	Forward:	1587-1606	5'-CGCTTCACCTCTGCACG-3'	492 bp
	Reverse:	2078-2062	5'-ATAGCTTGCCTGAGTGC-3'	
g.	Forward:	1956-1974	5'-TGCCTTCTGACTTCTTTCC-3'	530 bp
	Reverse:	2461-2485	5'-AGTTTCCCACCTTATGAGTC-3'	
h.	Forward:	2358-2376	5'-GCAGGTCCCCTAGAAGAAG-3'	534 bp
	Reverse:	2872-2891	5'-GTCCCCATGCCTTKTCGAGG-3'	
i	Forward:	2825-2845	5'-ATTCTTGGGAACAMGAKCTAC-3'	466 bp

Reverse:	76-62	5’-TGAACTGGAGCCACCAG-3’

IUPAC nucleotide code: W = base A or T, Y = base C or T, M = A or C, K = G or T.

Table S2. Mapping NGS datasets of genotype B patients (n = 34) to different references of HBV full genome.

Variables	JN315779 (Geno. C, Asia)	KJ790199 (Geno. C, Taiwan)	FJ787477 (Geno. B, Asia)	KJ790200 (Geno. B, Taiwan)	Sample-specific reference
Mappable reads (%)	87.18 ± 11.88***	87.26 ± 11.85***	87.55 ± 12.06***	88.08 ± 11.76***[a]	88.54 ± 11.85
Properly paired reads (%)	84.76 ± 11.71***	84.89 ± 11.72**	85.04 ± 11.82***	85.61 ± 11.50*	86.14 ± 11.58
Broken paired reads (%)	1.01 ± 0.63 ***	0.93 ± 0.65***	1.50 ± 0.97	1.52 ± 0.97*	1.54 ± 1.00
Singleton (%)	1.41 ± 1.02***	1.44 ± 1.00***	1.01 ± 0.87***	0.95 ± 0.83***	0.86 ± 0.78
Minimum Coverage per nucleotide	96 ± 254***	121 ± 252***	1199 ± 1808	1241 ± 1800	1248 ± 1798
Maximum Coverage per nucleotide	92767 ± 56992***	92889 ± 57160***	94188 ± 57581	94069 ± 57610	94744 ± 58080
Average Coverage per nucleotide	24340 ± 17197***	24221 ± 17101***	26475 ± 18788***	26547 ± 18816***[a]	26710 ± 18841
Nucleotides covered <30 (%)	0.71 ± 0.66***	0.33 ± 0.50***	0.03 ± 0.12	0.02 ± 0.11	0.02 ± 0.11
Nucleotides covered >1000 (%)	95.50 ± 4.14***	95.37 ± 4.44***	97.18 ± 4.34	97.21 ± 4.37*	97.31 ± 4.30

Total reads after quality trimming = 1,027,778 ± 615,106; data are mean ± standard deviation; JN315779, KJ790199, FJ787477, and KJ790200 were from GenBank database; Geno., genotype; sample-specific reference was from the NGS reads aligned to FJ787477; *P*-values for differences between samples-specific reference and each reference from the GenBank database (**P*<0.05; ***P*<0.01; ****P*<0.001) and for differences between FJ787477 and KJ790200 ([a]*P*<0.01) are from two-tailed independent *t* tests.

Table S3. Mapping results of simulating NGS reads from 158 HBV strains in NCBI GenBank with alignment to each strain of them.

	Mapping references[1]							
Subgenotype (no.)	A1 (17)	A2 (17)	B1 (7)	B2 (26)	C1 (13)	C2 (26)	D1 (26)	D2 (26)
Mappable reads/ total reads (%)								
A1 (17)	**99.9 ± 0.0**	99.6 ± 0.2	92.1 ± 3.5	91.7 ± 2.3	90.7 ± 2.5	91.7 ± 1.7	84.5 ± 2.8	86.0 ± 2.5
A2 (17)	99.6 ± 0.2	**99.9 ± 0.2**	92.6 ± 3.4	92.7 ± 1.7	88.6 ± 2.5	89.7 ± 1.4	87.3 ± 1.5	89.3 ± 1.6
B1 (7)	92.2 ± 3.3	92.6 ± 3.2	**95.5 ± 4.7**	95.8 ± 4.3	91.2 ± 4.4	91.7 ± 4.0	87.4 ± 4.9	88.1 ± 4.9
B2 (26)	91.6 ± 2.4	92.6 ± 1.6	94.9 ± 5.2	**99.8 ± 0.4**	93.1 ± 2.6	93.2 ± 2.0	86.0 ± 1.9	86.3 ± 2.2
C1 (13)	90.5 ± 2.5	88.4 ± 2.5	91.2 ± 4.4	93.1 ± 2.6	**98.8 ± 1.6**	98.7 ± 1.7	84.6 ± 2.1	84.9 ± 2.4
C2 (26)	91.8 ± 1.8	89.8 ± 1.4	91.9 ± 4.0	93.5 ± 1.9	98.8 ± 2.5	**99.7 ± 0.7**	83.4 ± 1.8	85.1 ± 2.1
D1 (26)	85.9 ± 2.6	88.4 ± 1.6	88.7 ± 4.6	87.8 ± 1.9	85.9 ± 2.2	84.8 ± 1.8	**99.7 ± 0.4**	99.8 ± 0.3
D2 (26)	86.8 ± 2.4	90.6 ± 1.0	88.8 ± 4.8	87.4 ± 2.3	85.6 ± 2.3	85.8 ± 2.0	99.7 ± 0.5	**99.8 ± 0.3**
Properly paired reads/ mappable reads (%)								
A1 (17)	**99.9 ± 0.0**	99.8 ± 0.1	97.7 ± 2.2	98.1 ± 0.7	96.6 ± 1.2	97.5 ± 1.1	95.9 ± 1.4	96.4 ± 1.0
A2 (17)	99.8 ± 0.1	**99.9 ± 0.0**	97.2 ± 0.8	98.2 ± 0.6	96.6 ± 1.3	96.9 ± 1.1	96.0 ± 0.7	96.8 ± 0.6
B1 (7)	97.8 ± 1.0	97.9 ± 0.8	**97.8 ± 1.0**	97.9 ± 1.6	96.1 ± 2.4	96.5 ± 1.9	95.1 ± 2.0	95.7 ± 1.8
B2 (26)	98.1 ± 0.7	98.2 ± 0.5	97.8 ± 1.8	**99.8 ± 0.4**	98.1 ± 0.8	97.5 ± 0.8	94.8 ± 1.3	95.2 ± 1.0
C1 (13)	96.6 ± 1.2	96.7 ± 1.3	96.1 ± 2.4	98.1 ± 0.8	**99.6 ± 0.7**	99.2 ± 0.8	92.7 ± 2.1	93.1 ± 1.8
C2 (26)	97.6 ± 1.1	97.2 ± 1.0	96.6 ± 1.8	97.6 ± 0.7	99.3 ± 0.7	**99.7 ± 0.6**	93.2 ± 1.5	94.1 ± 1.1
D1 (26)	96.3 ± 1.2	96.6 ± 0.5	95.7 ± 1.8	95.7 ± 0.8	93.6 ± 1.8	93.7 ± 1.2	**99.8 ± 0.4**	99.8 ± 0.3
D2 (26)	96.5 ± 1.0	97.1 ± 0.5	95.9 ± 1.7	95.7 ± 0.9	93.5 ± 1.8	94.3 ± 1.1	99.8 ± 0.5	**99.8 ± 0.3**

Singleton reads/ mappable reads (%)

A1 (17)	**0.0 ± 0.0**	0.1 ± 0.1	2.2 ± 2.2	1.8 ± 0.7	3.3 ± 1.2	2.4 ± 1.1	4.0 ± 1.4	3.5 ± 1.0
A2 (17)	0.1 ± 0.1	**0.0 ± 0.0**	2.7 ± 0.8	1.7 ± 0.6	3.3 ± 1.3	3.0 ± 1.1	3.9 ± 0.7	3.1 ± 0.6
B1 (7)	2.1 ± 1.0	2.0 ± 0.8	**2.1 ± 1.0**	2.0 ± 1.6	3.8 ± 2.4	3.4 ± 1.9	4.8 ± 2.0	4.2 ± 1.8
B2 (26)	1.8 ± 0.7	1.8 ± 0.5	2.1 ± 1.8	**0.1 ± 0.4**	1.8 ± 0.8	2.4 ± 0.8	5.2 ± 1.3	4.8 ± 1.0
C1 (13)	3.3 ± 1.2	3.2 ± 1.3	3.8 ± 2.4	1.8 ± 0.8	**0.3 ± 0.7**	0.7 ± 0.8	7.2 ± 2.1	6.8 ± 1.8
C2 (26)	2.3 ± 1.1	2.7 ± 1.0	3.3 ± 1.8	2.3 ± 0.7	0.6 ± 0.7	**0.2 ± 0.6**	6.7 ± 1.5	5.8 ± 1.1
D1 (26)	3.6 ± 1.2	3.3 ± 0.5	4.2 ± 1.8	4.2 ± 0.8	6.3 ± 1.8	6.2 ± 1.2	**0.1 ± 0.4**	0.1 ± 0.3
D2 (26)	3.4 ± 1.0	2.8 ± 0.5	4.0 ± 1.7	4.2 ± 0.9	6.4 ± 1.8	5.6 ± 1.1	0.2 ± 0.5	**0.1 ± 0.3**

[1]Each HBV full genome sequence of 158 strains was all used as mapping references for 158 simulating database produced from 158 HBV full genomes separated into different genotypes/subgenotypes.

Table S4. Prevalence of probable false SNVs of genotype C patients (n = 52) to different references of HBV full genome.

NT	Mapping reference (Geno. C/B)	Inconsistent SNVs comprising derived consensus sequences: JN315779 → FJ787477 (Geno. C, Asia) (Geno. B, Asia)	Number of patients (%)
		**C → A**	**2 (3.8)**
		A → A	14 (26.9)
		C → C	36 (69.2)
**85**	**A/G**	**A → G**	**2 (3.8)**
		A → A	50 (96.2)
**87**	**A/G**	**A → G**	**2 (3.8)**
		A → A	50 (96.2)
**93**	**T/C**	**T → C**	**2 (3.8)**
		T → T	50 (96.2)
**96**	**C/A**	**C → A**	**2 (3.8)**
		C → C	50 (96.2)
**99**	**C/A**	**C → A**	**2 (3.8)**
		C → C	50 (96.2)
**834**	**G/A**	**A → G**	**1 (1.9)**
		G → G	15 (28.8)
		A → A	36 (69.2)
**929**	**T/A**	**T → A**	**1 (1.9)**
		A → A	11 (21.2)
		T → T	40 (76.9)
**939**	**G/A**	**G → A**	**38 (73.1)**
		A → A	12 (23.1)
		G → G	2 (3.8)
**940**	**C/A**	**C → A**	**38 (73.1)**
		G → G	1 (1.9)
		A → A	1 (1.9)
		C → C	12 (23.1)
**941**	**A/T**	**A → T**	**27 (51.9)**
		A → A	25 (48.1)
**942**	**A/G**	**A → G**	**23 (44.2)**
		C → C	1 (1.9)
		T → T	1 (1.9)
		A → A	27 (51.9)
**949**	**C/A**	**C → A**	**1 (1.9)**
		A → A	2 (3.8)
		C → C	49 (94.2)
**951**	**A/G**	**C → G**	**2 (3.8)**
		C → C	3 (5.8)
		G → G	7 (13.5)

		T → T	6 (11.5)
		A → A	34 (65.4)
**957**	**G/T**	**G → T**	**2 (3.8)**
		T → T	20 (38.5)
		G → G	30 (57.7)
**969**	**A/G**	**A → G**	**1 (1.9)**
		G → G	3 (5.8)
		A → A	48 (92.3)
**1287**	**A/C**	**A → C**	**2 (3.8)**
		C → C	19 (36.5)
		A → A	31 (59.6)
**1306**	**C/T**	**C → A**	**1 (1.9)**
		A → A	4 (7.7)
		C → C	47 (90.4)
**1314**	**A/G**	**A → G**	**3 (5.8)**
		G → G	11 (21.2)
		A → A	38 (73.1)
**1317**	**G/A**	**G → A**	**2 (3.8)**
		A → A	18 (34.6)
		G → G	32 (61.5)
**1320**	**A/A**	**A → C**	**1 (1.9)**
		C → C	6 (11.5)
		A → A	45 (86.5)
**1323**	**T/C**	**T → C**	**3 (5.8)**
		C → C	21 (40.4)
		T → T	28 (53.8)
**1329**	**G/G**	**A → G**	**2 (3.8)**
		C → C	6 (11.5)
		G → G	4 (7.7)
		A → A	40 (76.9)
**1332**	**T/T**	**C → T**	**2 (3.8)**
		T → T	5 (9.6)
		C → C	45 (86.5)
**1338**	**C/T**	**C → T**	**6 (11.5)**
		T → T	3 (5.8)
		C → C	41 (78.8)
**1341**	**T/T**	**C → T**	**1 (1.9)**
		C → C	2 (3.8)
		T → T	49 (94.2)
**1344**	**T/C**	**T → C**	**7 (13.5)**
		C → C	3 (5.8)
		T → T	42 (80.8)
**1347**	**C/G**	**C → G**	**10 (19.2)**
		G → G	1 (1.9)

		C → C	41 (78.8)
**1353**	**T/C**	**T → C**	**15 (28.8)**
		C → C	1 (1.9)
		T → T	36 (69.2)
**1356**	**G/C**	**G → C**	**15 (28.8)**
		C → C	3 (5.8)
		G → G	34 (65.4)
**1359**	**A/G**	**A → G**	**13 (25.0)**
		G → G	1 (1.9)
		A → A	38 (73.1)
**1362**	**C/T**	**C → T**	**12 (23.1)**
		C → C	40 (76.9)
**1365**	**C/A**	**C → A**	**8 (15.4)**
		A → A	2 (3.8)
		C → C	42 (80.8)
**1368**	**C/A**	**C → A(3)/G(3)**	**6 (11.5)**
		T → T	2 (3.8)
		G → G	1 (1.9)
		C → C	43 (82.7)
**1461**	**G/C**	**G → C**	**3 (5.8)**
		C → C	4 (7.7)
		G → G	45 (86.5)
**1464**	**T/C**	**T → C**	**3 (5.8)**
		C → C	3 (5.8)
		T → T	46 (88.5)
**1473**	**T/C**	**T → C**	**3 (5.8)**
		C → C	5 (9.6)
		T → T	44 (84.6)
**1479**	**C/G**	**C → G**	**3 (5.8)**
		A → A	15 (28.8)
		C → C	12 (23.1)
		T → T	2 (3.8)
		G → G	20 (38.5)
**1488**	**T/C**	**T → C**	**5 (9.6)**
		C → C	2 (3.8)
		T → T	45 (86.5)
**1491**	**C/G**	**C → G**	**5 (9.6)**
		G → G	2 (3.8)
		T → T	2 (3.8)
		C → C	43 (82.7)
**1497**	**T/C**	**G(7)/T(2) → C**	**9 (17.3)**
		G → G	7 (13.5)
		C → C	2 (3.8)
		A → A	2 (3.8)

		T → T	32 (61.5)
**1499**	**A/G**	**A(4)/C(4) → G**	**8 (15.4)**
		G → G	17 (32.7)
		A → A	27 (51.9)
**1500**	**T/C**	**T → C**	**9 (17.3)**
		C → C	1 (1.9)
		T → T	42 (80.8)
**1504**	**C/T**	**C → T**	**10 (19.2)**
		C → C	42 (80.8)
**1505**	**C/T**	**C → T**	**6 (11.5)**
		C → C	46 (88.5)
**1508**	**T/T**	**T → A**	**2 (3.8)**
		A → A	2 (3.8)
		T → T	48 (92.3)
**1512**	**G/A**	**G → A**	**1 (1.9)**
		A → A	3 (5.8)
		G → G	48 (92.3)
**1632**	**C/G**	**C → A**	**2 (3.8)**
		A → A	1 (1.9)
		G → G	1 (1.9)
		C → C	48 (92.3)
**1633**	**A/G**	**A → G**	**3 (5.8)**
		G → G	3 (5.8)
		A → A	46 (88.5)
**1634**	**G/G**	**A → G(2)/C(1)**	**3 (5.8)**
		G → G	49 (94.2)
**1635**	**G/A**	**G → A**	**3 (5.8)**
		A → A	3 (5.8)
		G → G	46 (88.5)
**1636**	**T/A**	**T → A**	**3 (5.8)**
		A → A	3 (5.8)
		C → C	5 (9.6)
		T → T	41 (78.8)
**1638**	**T/C**	**T → C**	**1 (1.9)**
		C → C	2 (3.8)
		T → T	49 (94.2)
**2009**	**C/C**	**C → A**	**5 (9.6)**
		T → T	4 (7.7)
		C → C	41 (78.8)
**2291**	**G/G**	**A → C**	**1 (1.9)**
		G → G	51 (98.1)
**2292**	**C/C**	**A → C**	**1 (1.9)**
		C → C	51 (98.1)
**2293**	**T/A**	**T → A**	**1 (1.9)**

		T → T	51 (98.1)
**2351**	**C/C**	**T → C**	**6 (11.5)**
		G → G	46 (88.5)
**2353**	**A/A**	**C → A**	**1 (1.9)**
		A → A	51 (98.1)
**2732**	**C/T**	**C → T**	**1 (1.9)**
		T → T	1 (1.9)
		C → C	50 (96.2)
**2733**	**A/C**	**A → C**	**7 (13.5)**
		C → C	1 (1.9)
		A → A	44 (84.6)
**2735**	**A/G**	**A → G**	**7 (13.5)**
		A → A	45 (86.5)
**2738**	**T/A**	**T → G**	**4 (7.7)**
		C → C	3 (5.8)
		T → T	45 (86.5)
**2739**	**A/C**	**A → C**	**2 (3.8)**
		A → A	50 (96.2)
**2741**	**G/A**	**G → A**	**6 (11.5)**
		A → A	1 (1.9)
		G → G	45 (86.5)
**2753**	**T/C**	**T → C**	**3 (5.8)**
		C → C	1 (1.9)
		T → T	48 (92.3)
**2759**	**G/T**	**G → T**	**2 (3.8)**
		T → T	1 (1.9)
		G → G	49 (94.2)
**2768**	**T/A**	**T → G**	**1 (1.9)**
		T → T	51 (98.1)
**2771**	**C/G**	**C → T**	**1 (1.9)**
		A → A	3 (5.8)
		T → T	8 (15.4)
		C → C	40 (76.9)
**2774**	**T/C**	**T → C**	**1 (1.9)**
		T → T	51 (98.1)
**2775**	**C/T**	**C → T**	**1 (1.9)**
		T → T	2 (3.8)
		C → C	49 (94.2)
**2783**	**G/A**	**G → A**	**1 (1.9)**
		G → G	51 (98.1)
**2790**	**A/T**	**A → T**	**1 (1.9)**
		A → A	51 (98.1)
**2792**	**T/C**	**T → C**	**1 (1.9)**
		T → T	51 (98.1)

**2797**	**C/T**	**A → G**	**1 (1.9)**
		A → A	3 (5.8)
		G → G	48 (92.3)
**2798**	**A/A**	**C → T**	**1 (1.9)**
		T → T	7 (13.5)
		C → C	44 (84.6)
**2889**	**G/A**	**A → G**	**1 (1.9)**
		A → A	3 (5.8)
		G → G	48 (92.3)
**2980**	**T/C**	**T → C**	**17 (32.7)**
		C → C	1 (1.9)
		T → T	34 (65.4)
**2985**	**C/T**	**C → T**	**2 (3.8)**
		C → C	50 (96.2)
**2988**	**C/G**	**C → G**	**18 (34.6)**
		C → C	34 (65.4)
**2989**	**A/C**	**A → C**	**20 (38.5)**
		A → A	32 (61.5)
**2997**	**T/C**	**T → C**	**25 (48.1)**
		T → T	27 (51.9)
**2998**	**C/C**	**C → A**	**22 (42.3)**
		C → C	30 (57.7)
**2999**	**A/C**	**A → C**	**2 (3.8)**
		C → C	7 (13.5)
		A → A	43 (82.7)
**3000**	**C/C**	**A(6)/T(2) → C**	**8 (15.4)**
		A → A	8 (15.4)
		T → T	2 (3.8)
		C → C	34 (65.4)
**3006**	**A/G**	**A → G**	**26 (50.0)**
		G → G	1 (1.9)
		A → A	25 (48.1)
**3009**	**G/C**	**G(29)/A(2) → C**	**31 (59.6)**
		G → G	21 (40.4)
**3012**	**A/T**	**A → C**	**31 (59.6)**
		A → A	21 (40.4)
**3013**	**A/T**	**A(3)/C(2) → T**	**5 (9.6)**
		C → C	1 (1.9)
		T → T	2 (3.8)
		A → A	44 (84.6)
**3014**	**A/G**	**A → G**	**6 (11.5)**
		G → G	2 (3.8)
		A → A	44 (84.6)
**3015**	**T/G**	**T → C(23)/G(17)**	**40 (76.9)**

		G → G	2 (3.8)
		T → T	10 (19.2)
**3016**	**C/A**	**C → A**	**9 (17.3)**
		C → C	43 (82.7)
**3021**	**A/G**	**A → G**	**1 (1.9)**
		A → A	51 (98.1)
**3026**	**C/T**	**C → T**	**1 (1.9)**
		T → T	22 (42.3)
		C → C	29 (55.8)

SNV, single nucleotide variation; NT, nucleotide; Geno., genotype.

Table S5. Prevalence of probable false SNVs of genotype B patients (n = 34) to different references of HBV full genome.

NT	Mapping reference (Geno. B/C)	Inconsistent SNVs comprising derived consensus sequences FJ787477 (Geno. B, Asia) → JN315779 (Geno. C, Asia)	Number of patients (%)
		**A → G**	**1 (2.9)**
		G → G	13 (38.2)
		A → A	20 (58.8)
**25**	**T/G**	**T → G**	**1 (2.9)**
		G → G	13 (38.2)
		A → A	20 (58.8)
**27**	**A/T**	**A → T**	**1 (2.9)**
		T → T	3 (8.8)
		A → A	30 (88.2)
**85**	**G/A**	**G → A**	**3 (8.8)**
		G → G	31 (91.2)
**87**	**G/A**	**G → A**	**3 (8.8)**
		G → G	31 (91.2)
**93**	**C/T**	**C → T**	**4 (11.8)**
		C → C	30 (88.2)
**96**	**A/C**	**A → C**	**4 (11.8)**
		A → A	30 (88.2)
**99**	**A/C**	**A → C**	**4 (11.8)**
		A → A	30 (88.2)
**105**	**T/C**	**T → C**	**3 (8.8)**
		T → T	31 (91.2)
**109**	**T/A**	**T → A**	**3 (8.8)**
		T → T	29 (85.3)
		C → C	1 (2.9)
		G → G	1 (2.9)
**110**	**G/C**	**G → C**	**3 (8.8)**
		G → G	29 (85.3)
		T → T	1 (2.9)
		A → A	1 (2.9)
**126**	**C/T**	**C → T**	**1 (2.9)**
		C → C	3 (8.8)
		T → T	30 (88.2)

**165**	**T/C**	**T → C**	**1 (2.9)**
		T → T	33 (97.1)
**166**	**C/A**	**C → A**	**1 (2.9)**
		A → A	1 (2.9)
		C → C	32 (94.1)
**167**	**G/A**	**G → A**	**1 (2.9)**
		A → A	2 (5.9)
		T → T	2 (5.9)
		G → G	29 (85.3)
**287**	**A/G**	**A → G**	**1 (2.9)**
		A → A	33 (97.1)
**724**	**C/T**	**C → T**	**1 (2.9)**
		C → C	33 (97.1)
**927**	**A/A**	**A → G**	**1 (2.9)**
		T → T	1 (2.9)
		C → C	1 (2.9)
		A → A	31 (91.2)
**928**	**C/C**	**C → T**	**1 (2.9)**
		C → C	33 (97.1)
**929**	**A/T**	**A → T**	**1 (2.9)**
		A → A	33 (97.1)
**939**	**A/G**	**A → C**	**1 (2.9)**
		A → A	33 (97.1)
**940**	**A/C**	**A → C(23)/G(1)**	**24 (70.6)**
		C → C	2 (5.9)
		A → A	8 (23.5)
**941**	**T/A**	**T → A**	**31 (91.2)**
		C → C	1 (2.9)
		T → T	2 (5.9)
**942**	**G/A**	**G → A**	**31 (91.2)**
		A → A	1 (2.9)
		G → G	2 (5.9)
**1314**	**G/A**	**G→ A**	**1 (2.9)**
		A → A	3 (8.8)
		G → G	30 (88.2)
**1317**	**A/G**	**A → G**	**1 (2.9)**
		G → G	10 (29.4)
		A → A	23 (67.6)

**1323**	**C/T**	**C → T**	**1 (2.9)**
		T → T	1 (2.9)
		C → C	32 (94.1)
**1338**	**T/C**	**T → C**	**2 (5.9)**
		C → C	1 (2.9)
		T → T	31 (91.2)
**1344**	**C/T**	**C → T**	**4 (11.8)**
		T → T	30 (88.2)
**1347**	**G/C**	**G → C**	**5 (14.7)**
		C → C	3 (8.8)
		G → G	26 (76.5)
**1353**	**C/T**	**C → T**	**7 (20.6)**
		T → T	1 (2.9)
		C → C	26 (76.5)
**1356**	**C/G**	**C → G**	**11 (32.4)**
		G→ G	1 (2.9)
		C → C	22 (64.7)
**1359**	**G/A**	**G → A**	**15 (44.1)**
		A → A	1 (2.9)
		G → G	18 (52.9)
**1362**	**T/C**	**T → C**	**14 (41.2)**
		C → C	1 (2.9)
		T → T	19 (55.9)
**1365**	**A/C**	**A → C**	**6 (17.6)**
		C → C	2 (5.9)
		A → A	26 (76.5)
**1368**	**A/C**	**A(9)/G(1) → C**	**10 (29.4)**
		G → G	8 (23.5)
		C → C	8 (23.5)
		A → A	8 (23.5)
**1461**	**C/G**	**C → G**	**1 (2.9)**
		G → G	2 (5.9)
		C → C	31 (91.2)
**1464**	**C/T**	**C → T**	**2 (5.9)**
		T → T	3 (8.8)
		C → C	29 (85.3)
**1479**	**G/C**	**G → A**	**1(2.9)**
		G → G	33 (97.1)

**1488**	**C/T**	**C → T**	**3 (8.8)**
		T → T	1 (2.9)
		C → C	30 (88.2)
**1491**	**G/C**	**G → C**	**3 (8.8)**
		C → C	1 (2.9)
		G → G	30 (88.2)
**1497**	**C/T**	**C → T**	**3 (8.8)**
		T → T	1 (2.9)
		C → C	30 (88.2)
**1499**	**G/A**	**G → A(3)/T(1)**	**4 (11.8)**
		A → A	1 (2.9)
		G → G	29 (85.3)
**1500**	**C/T**	**C → T**	**12 (35.3)**
		T → T	1 (2.9)
		C → C	21 (61.8)
**1503**	**G/G**	**A → G**	**3 (8.8)**
		C → G(2)/T(1)	3 (8.8)
		C → C	2 (5.9)
		G → G	26 (76.5)
**1504**	**T/C**	**T(10)/G(3) → C**	**13 (38.2)**
		C → C	4 (11.8)
		G → G	1 (2.9)
		A → A	1 (2.9)
		T → T	15 (44.1)
**1505**	**T/C**	**T → C**	**13 (38.2)**
		G → G	1 (2.9)
		C → C	1(2.9)
		T → T	19 (55.9)
**1508**	**T/T**	**A → T**	**4 (11.8)**
		T → T	2 (5.9)
		A → A	28 (82.4)
**1632**	**G/C**	**G → C**	**1 (2.9)**
		G → G	13 (38.2)
		T → T	1 (2.9)
		C → C	19 (55.9)
**1635**	**A/G**	**A → G**	**2 (5.9)**
		A → A	32 (94.1)
**1636**	**A/T**	**A → T**	**2 (5.9)**

		A → A	32 (94.1)
**2486**	**G/T**	**G → T**	**1 (2.9)**
		T → T	2 (5.9)
		G → G	31 (91.2)
**2504**	**G/T**	**G → T**	**1 (2.9)**
		T → T	2 (5.9)
		G → G	31 (91.2)
**2511**	**T/G**	**T → G**	**1 (2.9)**
		G → G	2 (5.9)
		T → T	31 (91.2)
**2512**	**G/T**	**G → T**	**1 (2.9)**
		T → T	2 (5.9)
		G → G	31 (91.2)
**2523**	**A/G**	**A → G**	**2 (5.9)**
		G → G	3 (8.8)
		A → A	29 (85.3)
**2525**	**A/G**	**A → G**	**1 (2.9)**
		C → A	1 (2.9)
		C → C	7 (20.6)
		T → T	3 (8.8)
		G → G	2 (5.9)
		A → A	21 (61.8)
**2537**	**T/C**	**T → C**	**2 (5.9)**
		T → T	32 (94.1)
**2540**	**T/C**	**T → C**	**2 (5.9)**
		T → T	32 (94.1)
**2547**	**G/A**	**G → C**	**1 (2.9)**
		A → A	2 (5.9)
		C → C	2 (5.9)
		G → G	29 (85.3)
**2666**	**T/C**	**T → C**	**1 (2.9)**
		C → C	1 (2.9)
		T → T	32 (94.1)
**2708**	**C/T**	**A → T**	**1 (2.9)**
		T → T	3 (8.8)
		A → A	30 (88.2)
**2711**	**G/A**	**G → A**	**1 (2.9)**
		A → A	2 (5.9)

		G → G	31 (91.2)
**2733**	**C/A**	**C → A**	**5 (14.7)**
		C → C	29 (85.3)
**2735**	**G/A**	**G → A**	**13 (38.2)**
		G → G	21 (61.8)
**2738**	**A/T**	**G → T**	**7 (20.6)**
		G → G	27 (79.4)
**2739**	**C/A**	**C → A**	**11 (32.4)**
		C → C	5 (14.7)
		A → A	18 (52.9)
**2741**	**A/G**	**A → G**	**8 (23.5)**
		A → A	26 (76.5)
**2753**	**C/T**	**C → T**	**7 (20.6)**
		T → T	3 (8.8)
		C → C	24 (70.6)
**2759**	**T/G**	**T → G**	**17 (50.0)**
		G → G	3 (8.8)
		T → T	14 (41.2)
**2760**	**G/T**	**T → G**	**1 (2.9)**
		T → T	33 (97.1)
**2768**	**A/T**	**G → T**	**15 (44.1)**
		G → G	19 (55.9)
**2771**	**G/C**	**G(10)/T(6) → C**	**16 (47.1)**
		G → G	6 (17.6)
		T → T	12 (35.3)
**2774**	**C/T**	**C → T**	**17 (50.0)**
		A → A	3 (8.8)
		C → C	14 (41.2)
**2775**	**T/C**	**T → C**	**17 (50.0)**
		C → C	1 (2.9)
		T → T	16 (47.1)
**2783**	**A/G**	**A → G**	**17 (50.0)**
		G → G	1 (2.9)
		A → A	16 (47.1)
**2789**	**G/A**	**G → A**	**15 (44.1)**
		G → G	19 (55.9)
**2790**	**T/A**	**T → A**	**16 (47.1)**
		A → A	3 (8.8)

		T → T	15 (44.1)
**2792**	**C/T**	**C → T**	**8 (23.5)**
		T → T	1 (2.9)
		A → A	1 (2.9)
		C → C	24 (70.6)
**2837**	**A/A**	**C → A**	**1 (2.9)**
		C → C	11 (32.4)
		A → A	22 (64.7)
**2840**	**T/G**	**T → G**	**1 (2.9)**
		T → T	16 (47.1)
		G → G	17 (50.0)
**2875**	**A/C**	**A → C**	**1 (2.9)**
		C → C	3 (8.8)
		A → A	30 (88.2)
**2901**	**C/T**	**C → T**	**1 (2.9)**
		T → T	2 (5.9)
		C → C	31 (91.2)
**2910**	**C/T**	**C → T**	**1 (2.9)**
		T → T	2 (5.9)
		C → C	31 (91.2)
**2922**	**C/T**	**C → T**	**1 (2.9)**
		T → T	4 (11.8)
		C → C	29 (85.3)
**2931**	**T/C**	**T → C**	**1 (2.9)**
		C → C	1 (2.9)
		T → T	32 (94.1)
**2946**	**A/G**	**A → G(1)/T(1)**	**2 (5.9)**
		G → G	2 (5.9)
		C → C	1 (2.9)
		A → A	29 (85.3)
**2950**	**A/G**	**A → G**	**2 (5.9)**
		A → A	32 (94.1)
**2951**	**A/G**	**A → G**	**3 (8.8)**
		G → G	2 (5.9)
		A → A	29 (85.3)
**2962**	**G/A**	**G → A**	**2 (5.9)**
		A → A	1 (2.9)
		G → G	31 (91.2)

**2964**	**A/C**	**A → C**	**2 (5.9)**
		C → C	3 (8.8)
		A → A	29 (85.3)
**2980**	**C/T**	**C → T**	**4 (11.8)**
		C → C	30 (88.2)
**2985**	**T/C**	**T → C**	**1 (2.9)**
		C → C	33 (97.1)
**2988**	**G/C**	**G(3)/A(1) → C**	**4 (11.8)**
		A → A	2 (5.9)
		G → G	28 (82.4)
**2989**	**C/A**	**C → A**	**5 (14.7)**
		C → C	29 (85.3)
**2997**	**C/T**	**C → T**	**4 (11.8)**
		C → C	30 (88.2)
**2998**	**C/C**	**A → C**	**4 (11.8)**
		A → A	30 (88.2)
**2999**	**C/A**	**G → A**	**2 (5.9)**
		G → G	1 (2.9)
		A → A	31 (91.2)
**3000**	**C/C**	**C → T**	**1 (2.9)**
		C → C	33 (97.1)
**3006**	**G/A**	**G → A**	**7 (20.6)**
		G → G	27 (79.4)
**3009**	**C/G**	**C → G**	**6 (17.6)**
		C → C	28 (82.4)
**3010**	**G/G**	**T → G**	**1 (2.9)**
		T → T	1 (2.9)
		G → G	32 (94.1)
**3012**	**T/A**	**C → A**	**6 (17.6)**
		C → C	28 (82.4)
**3013**	**T/A**	**T → A**	**1 (2.9)**
		A → A	33 (97.1)
**3014**	**G/A**	**G → A**	**1 (2.9)**
		A → A	33 (97.1)
**3015**	**G/T**	**C → T**	**5 (14.7)**
		C → C	29 (85.3)
**3016**	**A/C**	**A → C**	**4 (11.8)**
		A → A	30 (88.2)

**3021**	**G/A**	**G → A**	**2 (5.9)**
		G → G	32 (94.1)
**3026**	**T/C**	**T → C**	**2 (5.9)**
		T → T	32 (94.1)
**3033**	**A/A**	**G → A**	**1 (2.9)**
		G → G	2 (5.9)
		A → A	31 (91.2)
**3120**	**T/G**	**T → A**	**1 (2.9)**
		T → T	33 (97.1)

SNV, single nucleotide variation; NT, nucleotide; Geno., genotype.
